# Complications of pulsed-field ablation for atrial fibrillation: a narrative structured review

**DOI:** 10.3389/fcvm.2026.1884313

**Published:** 2026-07-22

**Authors:** Jiangyao Li, Jia Gao, Rui Wang

**Affiliations:** 1 First Clinical Medical College, Shanxi Medical University, Taiyuan, China; 2 Department of Cardiology, First Hospital of Shanxi Medical University, Taiyuan, Shanxi, China

**Keywords:** acute kidney injury, atrial fibrillation, complications, coronary spasm, haemolysis, irreversible electroporation, phrenic nerve injury, pulsed-field ablation

## Abstract

Pulsed-field ablation (PFA) has been adopted at remarkable speed as a non-thermal approach for pulmonary vein isolation in atrial fibrillation (AF). Its mechanism—irreversible electroporation—preferentially injures cardiomyocytes while theoretically sparing adjacent heat-sensitive structures. Landmark randomised trial data (ADVENT) and large prospective registries (MANIFEST-17K; MANIFEST-PF) confirm that PFA is non-inferior to conventional thermal ablation for arrhythmia freedom and carries an overall favourable safety profile, with particularly low rates of atrooesophageal fistula and pulmonary vein stenosis. Nonetheless, substituting electrical for thermal energy introduces a qualitatively distinct injury spectrum. Intravascular haemolysis, acute kidney injury, transient coronary vasospasm, phrenic nerve stimulation, silent cerebral emboli, and—importantly—the risk of ventricular fibrillation during unsynchronised pulse delivery have each been documented across prospective cohorts and multicentre registries. This review provides a narrative structured synthesis of mechanistic and clinical evidence for each PFA-associated complication, proposes a three-tier classification (PFA-specific, PFA-enriched, and general ablation-related), and outlines practical risk-mitigation strategies. Recognition that “non-thermal” does not equate to “risk-free” is essential as PFA transitions to standard of care.

## Introduction

Atrial fibrillation is the most common sustained cardiac arrhythmia worldwide, carrying independent risks of stroke, heart failure, and premature mortality (1). Data from the Framingham Heart Study documented a fourfold rise in AF prevalence and threefold increase in incidence over five decades (1). Catheter-based rhythm control, centred on pulmonary vein isolation (PVI), has become a cornerstone of management for symptomatic AF.

Thermal ablation platforms—radiofrequency current and cryoablation—have demonstrated durable efficacy but are associated with well-characterised collateral injuries. Atrooesophageal fistula, though rare, carries a mortality rate approaching 50% (2). Pulmonary vein stenosis and persistent phrenic nerve palsy are further manifestations of the fundamental inability of heat to discriminate between target myocardium and surrounding structures.

PFA represents a mechanistically distinct strategy utilising ultra-short, high-voltage pulses to induce irreversible electroporation—cell death via nanoscale transmembrane pore formation, without heat generation. Large registries and, critically, the ADVENT randomised controlled trial have established that PFA achieves non-inferior arrhythmia freedom relative to thermal ablation while largely eliminating thermal injury patterns (3, 4). This overall favourable profile has driven rapid global adoption. However, as experience has expanded, a qualitatively distinct complication spectrum has emerged, rooted in electrochemical, haematological, neurohumoral, and proarrhythmic mechanisms. Understanding these complications is essential for safe implementation of PFA at scale.

This review examines the mechanistic basis and clinical evidence for PFA-associated complications, evaluates the determinants of risk, and synthesises evidence-based mitigation strategies.

## Methods

This is a narrative structured review. A systematic literature search was performed in PubMed and Embase from database inception to May 2026. Search terms included: “pulsed field ablation,” “irreversible electroporation,” “atrial fibrillation ablation,” “complications,” “haemolysis,” “acute kidney injury,” “coronary artery spasm,” “phrenic nerve injury,” “cerebral embolism,” “ventricular fibrillation,” and “proarrhythmia.” Only publications in English were eligible. Results were supplemented by manual searching of reference lists and conference abstracts. Evidence was hierarchically weighted, prioritising randomised controlled trials and large prospective registries, followed by prospective cohort studies, mechanistic investigations, and case series where higher-level evidence was unavailable. This review did not include unpublished or personal communications. A formal PRISMA flowchart was not constructed because PRISMA guidance is designed for systematic reviews and meta-analyses that conform to a pre-registered, exhaustive retrieval protocol with quantitative evidence synthesis. The present work is intentionally a narrative structured review: it synthesises mechanistically heterogeneous evidence—spanning large randomised trials, prospective registries, cohort studies, mechanistic preclinical investigations, and case series—and provides clinical interpretation across these strata rather than fulfilling the methodological criteria for a systematic review. Readers are directed to the complication classification framework (below) for a transparent account of how evidence was organised and weighted.

### Complication classification framework

Informed by the available evidence, we propose a three-tier classification of PFA-associated complications:

1. PFA-specific complications—arising directly from the electroporative mechanism and not observed with thermal ablation: intravascular haemolysis and secondary acute kidney injury.
2. PFA-enriched complications—occurring at clinically meaningful rates with PFA but less frequent or mechanistically distinct from those of thermal ablation: coronary artery spasm, phrenic nerve capture, Takotsubo cardiomyopathy, and proarrhythmic ventricular fibrillation.
3. General ablation-related complications—shared across energy modalities, with incidence and precipitants that may differ: vascular access complications, cardiac tamponade, and silent cerebral embolic events.

This framework is applied throughout the following sections and is incorporated into Table 1.

**Table 1:** PFA complication overview with three-tier classification.

**Complication**	**Category**	**Incidence**	**Mechanism**	**Timing**	**Features**	**Mitigation**
Vascular complications	General	0.30%	Access-site injury	Perioperative	Swelling, pain	Refined access technique
Cardiac tamponade	General	0.36%	Perforation; trans-septal injury	Perioperative	Haemodynamic compromise	Minimise catheter force
Intravascular haemolysis	PFA-specific	>90% biochemical	Off-tissue discharge; high field; excess pulses	Intraoperative to hours post	Haemoglobinuria; elevated LDH	Optimise contact; titrate pulses
Acute kidney injury	PFA-specific	0.03–14.3%	Haemoglobin tubular injury; oxidative stress	Hours to days post	Oliguria, rising creatinine	Renal assessment; hydration
Coronary artery spasm	PFA-enriched	0.14%	Field-mediated smooth muscle depolarisation; may be diffuse	Intraoperative; delayed > 15–30 min post	Chest pain; ST changes; may occur with PVI only	Nitrate prophylaxis; ACT > 300 s; monitor ≥30 min post
Phrenic nerve injury	PFA-enriched	0.46% clinical; up to 40.6% monitored	Electrical capture	Intraoperative	Cough; dyspnoea	CMAP monitoring
Silent cerebral lesions	General	∼15% DWI-MRI	Micro-embolisation	Perioperative	Usually asymptomatic	ACT > 300 s; anticoagulation
Ventricular fibrillation	PFA-enriched	Rare; registry cases	Vulnerable-period delivery; synchronisation failure	Intraoperative	Sudden cardiac arrest; responds to defibrillation	R-wave synchronisation; confirm before each delivery; defibrillator ready
Takotsubo syndrome	PFA-enriched	Rare (case reports)	Autonomic surge	Within hours post	ST elevation; reversible LV dysfunction	ECG and echo monitoring

### Mechanistic foundations of PFA-related injury

#### Electroporation thresholds and tissue selectivity

The electroporation threshold—the field intensity at which transmembrane pore formation becomes irreversible—differs substantially between cell types (5). Cardiomyocytes reach this threshold at lower field intensities than vascular smooth muscle, fibroblasts, or neuronal structures, providing the theoretical basis for myocardial selectivity. In practice, however, this selectivity is conditional on electrode geometry, catheter angle, and proximity to target tissue. In thin-walled structures or near blood-filled cavities, non-target cells may be exposed to supraphysiological field intensities.

#### Erythrocyte vulnerability and haemolysis

Erythrocytes are susceptible to electroporation and undergo osmotic lysis when pore formation exceeds membrane repair capacity (6). Biochemical haemolysis—elevations in free haemoglobin, lactate dehydrogenase (LDH), and bilirubin—is near-universal across PFA platforms (7, 8). Key drivers include off-tissue discharge into the blood pool, high field strength, and large cumulative pulse numbers (9).

#### Vascular smooth muscle and coronary reactivity

Coronary smooth muscle cells appear more susceptible to field-mediated depolarisation than vascular endothelium. Acute vasospasm during PFA most plausibly reflects direct smooth muscle excitation. Pre-clinical data have additionally demonstrated neointimal hyperplasia and late luminal narrowing after epicardial PFA exposure (10–12), though the clinical relevance of endocardial application remains to be established by long-term surveillance.

#### Neural susceptibility and phrenic nerve stimulation

Phrenic nerve fibres occupy an intermediate position in the electroporation threshold hierarchy. Capture tends to occur when energy is delivered adjacent to the right-sided pulmonary veins or superior vena cava (4, 13, 14). PFA-induced phrenic dysfunction is typically transient (reversible membrane depolarisation) rather than permanent (axonal necrosis as seen with thermal injury) (15).

#### Cardiac synchronisation and proarrhythmic risk

Contemporary PFA systems deliver pulses synchronised to the cardiac cycle to avoid the ventricular vulnerable period (T wave). Delivery during the T wave carries the risk of inducing ventricular fibrillation (VF) through R-on-T phenomena. Biphasic pulse waveforms have been shown in pre-clinical models to carry a lower VF threshold than monophasic waveforms, which has informed current device design. Synchronisation failure, inappropriate sensing, or delivery during irregular rhythms represents a residual mechanistic risk. PFA has also been explored for ventricular tachycardia ablation, highlighting the complex interaction between electroporation and ventricular myocardium (16).

### Clinical complication profile

The following subsections are organised according to the three-tier classification framework described above. Table 1 provides a structured overview of each complication category.

#### General ablation-related: vascular complications and cardiac tamponade

The MANIFEST-17K study (17,642 patients) reported vascular complication and cardiac tamponade rates of 0.30% and 0.36%, respectively (3). In MANIFEST-PF (*n* = 1,758), minor vascular events occurred in 3.28% of procedures (4). Tamponade arose from catheter manipulation, trans-septal complications, and one case of steam micro-explosion (3). Catheter mechanical failures have also been documented (17).

#### PFA-specific: intravascular haemolysis

Biochemical haemolysis accompanies virtually every contemporary PFA procedure, scaling with pulse number and confirmed across catheter platforms (7, 8). Overt haemoglobinuria is infrequent; transient bilirubin elevation is common. The clinical relevance in healthy patients is limited, but those with pre-existing renal impairment require enhanced surveillance.

#### PFA-specific: acute kidney injury

When haemolytic output exceeds haptoglobin scavenging capacity, free haemoglobin reaches the glomerular filtrate, causing pigment nephropathy, oxidative tubular damage, and vasoconstriction via nitric oxide scavenging (6). Reported AKI incidence ranges from 0.03% to 14.3% (6). In MANIFEST-17K, five patients (0.03%) required dialysis (3). Most cases resolved with hydration (18). Pre-procedural renal assessment and structured perioperative hydration are standard practice.

#### PFA-enriched: coronary artery spasm

Coronary vasospasm is enriched with PFA compared with thermal ablation platforms, where it has been reported but is considerably less frequent (3, 4). In the thermal ablation literature, vasospasm has occasionally been documented with cryoballoon and radiofrequency energy, but the mechanism—predominantly mechanical or thermal irritation—is distinct from the field-mediated smooth muscle depolarisation that underlies PFA-associated spasm.

Clinically, vasospasm manifests most commonly during ablation near the mitral isthmus or inferior vena cava–tricuspid annulus junction. In MANIFEST-17K, 0.14% of patients developed angiographically confirmed spasm (3).

Importantly, vasospasm may present in a delayed fashion, occurring 15–30 or more minutes after the last pulse delivery. This delayed pattern may reflect sustained smooth muscle reactivity, vagal rebound, or inflammatory mediator release following electroporation. Operators should therefore maintain ECG monitoring and vasodilator availability for at least 30 min beyond the conclusion of energy delivery, and post-procedural chest pain or ST-segment changes should prompt urgent coronary evaluation regardless of the interval since ablation.

Vasospasm has also been reported during PVI-only procedures, suggesting that the mechanism can be diffuse or systemic rather than localised to the catheter tip. Proposed explanations include release of vasoactive substances from pulmonary vein myocardium during electroporation, systemic autonomic activation, or micro-embolic phenomena affecting distal coronary vasculature. This observation has implications for risk stratification: patients with pre-existing coronary artery disease or vasospastic angina warrant particular vigilance even when ablation is confined to the pulmonary veins.

Regarding procedural management, ACT should be maintained above 300 s throughout all left atrial access procedures, and meticulous air purging of transseptal sheaths and delivery systems is mandatory, as intracardiac air can precipitate or closely mimic vasospasm clinically and electrocardiographically. Malyshev et al. evaluated two prophylactic nitroglycerin strategies—serial intra-right-atrial bolus injections vs. a combined bolus-plus-infusion regimen—and reported acceptable safety signals for both, though head-to-head comparative data and optimal dosing protocols have yet to be defined (19). Whether routine pre-procedural coronary angiography should be performed remains unsettled; current practice supports selective angiography in patients with known or suspected coronary artery disease, exertional symptoms, or a history of vasospastic angina, while most operators defer to on-demand evaluation triggered by intraoperative ST-segment changes or haemodynamic compromise in otherwise low-risk patients. The frequency of angiographically confirmed vasospasm is substantially lower with radiofrequency and cryoballoon platforms—where spasm, when reported, most plausibly reflects catecholamine release or mechanical irritation rather than direct field-mediated smooth-muscle depolarisation—reinforcing the mechanistic distinction between PFA-associated spasm and thermal ablation-related events. This relative enrichment of vasospasm with PFA justifies its classification as a PFA-enriched, rather than a general ablation-related, complication.

#### PFA-enriched: phrenic nerve injury

MANIFEST-PF recorded transient phrenic nerve paralysis in 0.46% (4). Chéhirlian et al. reported an incidence of 40.6% with dedicated monitoring, occurring at initial contact in nearly half of affected patients (15). CMAP monitoring detected impending injury in 21.9%, and voltage thresholds ranged from 0.3 to 0.8 V in the inferior vena cava to 1.3–2.8 V in the superior vena cava (20).

#### General ablation-related: silent cerebral events and stroke

Diffusion-weighted MRI has detected acute silent cerebral lesions in approximately 15% of PFA patients (21). Two distinct embolic mechanisms contribute to this burden. The first is thromboembolic: catheter manipulation within the left atrium, transseptal instrumentation, and prolonged sheath dwell time all create conditions for clot formation, and any lapse in anticoagulation substantially amplifies risk. The second mechanism is unique to PFA: electrolytic gas generation at the electrode-blood interface produces hydrogen and oxygen microbubbles during high-voltage pulse delivery, and these submillimetre particles can traverse the pulmonary circulation and enter the cerebral vasculature before being absorbed. Uninterrupted periprocedural oral anticoagulation—without bridging interruption—combined with an intraoperative ACT target above 300 s throughout left atrial dwell time constitutes the primary preventive strategy (4). Bubble management is an additional consideration: frequent aspiration of the delivery catheter lumen, meticulous de-airing of transseptal sheaths, and avoiding unnecessary contrast injections near the pulmonary veins may help reduce gas-bubble embolic load, though standardised protocols for bubble prevention specific to PFA have not yet been established in prospective trials. DWI-MRI studies specifically designed to evaluate PFA have documented silent cerebral lesions or events in a subset of patients, with microbubble generation at the electrode–blood interface during electrolysis identified as a contributing embolic source distinct from conventional thromboembolic pathways (22). The neurological assessment subgroup of the ADVENT randomised trial—the first prospective, head-to-head comparison of cerebral outcomes between PFA and thermal ablation—detected three subclinical brain lesions in the PFA arm vs. none in the thermal arm within a small MRI-assessed cohort, with no clinical neurological deficits in either group, suggesting broadly comparable cerebral safety across modalities (23).

#### PFA-enriched: proarrhythmic risk and ventricular fibrillation

High-voltage pulse delivery during PFA carries a risk of inducing ventricular fibrillation (VF) if pulses are delivered during the ventricular vulnerable period. Contemporary PFA systems mitigate this through R-wave synchronised delivery; however, synchronisation failures, sensing errors during arrhythmic rhythms, or system-specific programming limitations may result in vulnerable-period delivery.

In large registries, VF during PFA has been documented as an infrequent but serious complication. MANIFEST-17K records isolated VF events; pre-clinical studies confirm that biphasic waveforms carry a lower VF induction threshold than monophasic waveforms, which informed current catheter design priorities. Non-PVI ablation targets—including the posterior wall, cavotricuspid isthmus, and superior vena cava—may carry elevated VF risk due to greater proximity to ventricular myocardium.

Clinical risk mitigation requires: confirmation of cardiac synchronisation before each energy delivery; continuous ECG monitoring with immediately available defibrillation; avoidance of PFA during uncontrolled atrial or ventricular arrhythmias unless the delivery system is specifically validated for this context; and operator familiarity with system-specific synchronisation algorithms. VF during PFA is generally immediately responsive to defibrillation, but even a brief VF episode in a haemodynamically compromised patient carries risk.

The dual character of PFA—simultaneously antiarrhythmic and potentially proarrhythmic—reflects the spatial gradient of electric field intensity inherent to catheter-based delivery. Tissue in direct contact with the active electrode receives field intensities sufficient to produce irreversible electroporation and permanent cardiomyocyte death; at increasing distances, the attenuated field generates only transient, sublethal membrane permeabilisation that resolves without structural injury. This transition zone between irreversible and reversible electroporation creates spatially heterogeneous electrical properties across adjacent myocardial sites, which may provide a substrate for re-entrant excitation and triggered arrhythmia. The same spatial gradient also has implications for ablation dose optimisation: reducing the vulnerable transition zone may narrow the window for proarrhythmic substrate formation. A pharmacological approach under investigation involves pre-procedural intra-arterial lidocaine delivery, which lowers the electroporation threshold such that the IRE lesion zone expands while reversible permeabilisation is reduced, with a corresponding attenuation of ventricular arrhythmia incidence in preclinical models (24). Whether systemic intravenous lidocaine achieves equivalent threshold modulation, and how this pharmacodynamic effect interacts with different waveform designs or energy settings, remain unresolved questions that will require prospective evaluation.

#### PFA-enriched: Takotsubo cardiomyopathy and rare systemic events

Isolated case reports describe Takotsubo cardiomyopathy following PFA (25). Intense autonomic activation accompanying high-voltage delivery may precipitate apical ballooning in susceptible individuals. The rarity precludes reliable incidence estimation.

### Procedural and patient-level risk determinants

#### Electrode-tissue proximity and contact quality

Groen et al. showed that higher tissue proximity index values produced deeper lesions (1.5 ± 0.5 vs. 1.3 ± 0.5 mm; *P* = 0.016) and larger surface areas (8.3 ± 2.9 vs. 6.4 ± 2.7 mm^2^; *P* < 0.001) in 162 experimental lesions (26). Howard et al. quantified a linear inverse relationship between electrode-epicardial distance and lesion depth (27). Poor apposition simultaneously reduces efficacy and worsens haemolysis by directing energy into the blood pool (28). ICE-guided contact verification before each delivery is now near-essential practice (29).

#### Pulse parameters: field strength and application number

Haemolysis severity scales with pulse frequency (18) and field strength (30, 31). Osmancik et al. found significant haemolysis above 54 Farawave discharges per procedure (32). Pulse-minimisation protocols are supported by these converging data.

#### Baseline renal function

Patients with pre-procedural eGFR < 50 mL/min/1.73 m^2^, large pulse burden, procedural dehydration, or prolonged procedure duration are at highest AKI risk. Pre-procedural renal assessment and perioperative hydration are standard mitigation. Evidence-based risk-mitigation strategies for each complication category are summarised in Table 2.

**Table 2:** Practical risk-reduction strategies.

No. Evidence-based risk-reduction strategy
Verify electrode-tissue contact with intracardiac echocardiography before each energy delivery. Keep ACT above 300 s throughout; do not interrupt anticoagulation. Minimise total pulse number: use the fewest applications that achieve durable isolation. Ensure adequate perioperative hydration, particularly in patients with reduced baseline renal function. Use CMAP monitoring during ablation near the right-sided pulmonary veins and superior vena cava. Administer nitrate prophylaxis selectively when working near the mitral isthmus, tricuspid annulus, or inferior caval junction. Confirm cardiac synchronisation before each PFA delivery; ensure defibrillation capability is immediately available throughout the procedure. Maintain ECG and haemodynamic monitoring for at least 30 min after the last energy delivery to detect delayed coronary vasospasm. Perform pre-procedural coronary evaluation and apply heightened vigilance in patients with known coronary artery disease or vasospastic angina, including during PVI-only procedures.

## Discussion

PFA has eliminated the most feared thermal injury patterns while introducing a distinct complication landscape. Registry and randomised trial data now provide a sufficiently robust evidence base to characterise both the overall safety advantages of PFA and its category-specific risks.

### Comparative efficacy and safety: the ADVENT trial and beyond

The ADVENT randomised controlled trial [Reddy et al. (33)] was the first prospective, multicentre RCT to directly compare PFA with conventional thermal ablation (radiofrequency or cryoballoon) for paroxysmal AF. PFA was non-inferior to thermal ablation for freedom from arrhythmia recurrence at 12 months (73.3% vs. 71.3%; posterior probability of non-inferiority 0.999), with a favourable procedural safety profile and shorter procedure times (33). The four-year ADVENT-LTO follow-up [Reddy et al. (34)] demonstrated preserved effectiveness over the long term, with a trend favouring PFA for freedom from hospital-based arrhythmia intervention (85.6% vs. 78.6%) and fewer repeat ablations (10.4% vs. 17.7%) (34). A network meta-analysis by Mariani et al. (35) comparing pentaspline PFA with high-power short-duration and very high-power short-duration radiofrequency ablation reported similar efficacy and overall safety profiles, with shorter procedure times for PFA (35). Taken together, these data unambiguously position PFA as an experientially validated, guideline-relevant therapeutic option—not merely a promising experimental technique. The ADVENT trial in particular is pivotal: it provides the level-1 randomised evidence that transforms PFA from registry-documented to rigorously proven, and its favourable four-year durability data further substantiate PFA as a standard-of-care alternative to established thermal platforms. Earlier single-arm multicentre programmes — IMPULSE, PEFCAT, and PEFCAT II — provided the foundational safety and efficacy data that preceded the randomised era (36).

Several fundamental questions remain unanswered. Whether biochemical haemolysis magnitude predicts long-term renal functional decline requires dedicated collaborative study. Longitudinal coronary imaging is needed to determine whether repeated smooth muscle depolarisation carries any late remodelling risk. The clinical relevance of silent MRI lesions for neurocognitive outcomes is unquantified. Post-market surveillance registries with standardised complication definitions are the essential infrastructure for resolving these questions.

Optimising pulse protocols, refining dosimetric thresholds, and building better synchronisation algorithms will define the next PFA development cycle. Clinicians must internalise that “non-thermal” is not “complication-free,” and that continued vigilance will be as important as procedural efficiency as this technology matures.

## Conclusions

PFA has transformed the safety landscape of AF ablation by eliminating the principal thermal injury risks while delivering non-inferior arrhythmia outcomes. Its adoption has concurrently unveiled a distinct complication spectrum, herein classified as PFA-specific (haemolysis, AKI), PFA-enriched (coronary vasospasm, phrenic nerve injury, proarrhythmia, Takotsubo), and general ablation-related (vascular access complications, tamponade, cerebral emboli). Most events are self-limiting and respond to targeted measures, but mechanistic understanding, dose-response characterisation, and long-term outcome data remain incomplete. Systematic mitigation—contact optimisation, pulse minimisation, anticoagulation, CMAP monitoring, nitrate prophylaxis, and synchronisation vigilance—constitutes the current evidence base. “Non-thermal” is not “complication-free,” and continued vigilance is indispensable.
